# MSC–Hydrogel Composite Systems for Knee Cartilage Repair and Osteoarthritis: A Systematic Review

**DOI:** 10.3390/gels12080715

**Published:** 2026-08-13

**Authors:** Yerik Raimagambetov, Birzhan Suiindik, Meruyert Makhmetova, Dina Saginova, Ulunay Kanatli, Gulzhanat Korganbekova

**Affiliations:** 1 Department of Orthopedics, National Scientific Center of Traumatology and Orthopedics Named After Academician N.D. Batpenov, Astana 010000, Kazakhstan; raymagambetov@list.ru (Y.R.); saginova_d@nscto.kz (D.S.);; 2 Institute of Life Sciences, Karaganda Medical University, Karaganda 100000, Kazakhstan; 3 School of Medicine, Astana Medical University, Astana 010000, Kazakhstan; 4 Department of Orthopedics and Traumatology, School of Medicine, Gazi University, Ankara 06560, Türkiye

**Keywords:** mesenchymal stem cells, hydrogel, knee cartilage, osteoarthritis, systematic review, GRADE

## Abstract

**Background:** MSC–hydrogel composite systems were developed to overcome the poor cell retention and limited durability of conventional marrow stimulation and suspension-based MSC delivery. A rigorous synthesis of the clinical evidence is lacking. **Objectives:** To evaluate the safety and efficacy of MSC–hydrogel composite therapy for focal knee cartilage defects and knee osteoarthritis, and to assess the certainty of evidence using the GRADE framework. **Methods:** PROSPERO-registered systematic review (CRD420261393525), conducted and reported per PRISMA 2020 and SWiM. Five databases (Embase, PubMed/MEDLINE, Cochrane CENTRAL, Scopus, Web of Science) were searched in May 2026 without date, language, or design restrictions. Adults receiving MSCs co-delivered in a hydrogel carrier for knee cartilage pathology were eligible. Risk of bias was assessed using RoB 2 (RCTs) and ROBINS-I (non-randomized studies). Narrative synthesis following Popay et al. was the primary method; GRADE certainty was assessed per outcome domain. **Results:** Ten studies (***N*** = 521) were identified. Eight studies fulfilled the predefined eligibility criteria for MSC–hydrogel composite interventions and formed the primary evidence synthesis. Two additional studies were retained as contextual comparators because they evaluated either hydrogel-based therapy without MSC administration or MSC therapy without a structured hydrogel carrier. Surgical MSC–hydrogel implantation was associated with improvements in cartilage repair. Intra-articular injection without a hydrogel scaffold produced synovitis reduction but no detectable structural regeneration at six months. No serious treatment-related adverse events were recorded. Risk of bias was serious or critical in seven of nine assessable studies; GRADE certainty was low to very low across all outcome domains. **Conclusions:** MSC–hydrogel composite implantation may provide favorable safety signals and directionally positive effects on cartilage repair, pain, and function, with seven-year follow-up data suggesting a possible durability advantage over marrow stimulation. However, adverse-event reporting was inconsistent, and certainty of evidence remains low or very low because most studies were non-randomized, single-center, and concentrated around one commercial platform. These findings are relevant to international cartilage-regeneration research because they identify key methodological limitations and trial-design priorities for translating MSC–hydrogel systems across different clinical and regulatory settings.

## 1. Introduction

Articular cartilage is avascular, aneural, and has severely limited intrinsic self-repair capacity. Focal chondral and osteochondral knee defects from acute injury, mechanical overload, and the progressive degeneration characteristic of osteoarthritis tend to enlarge over time and represent a recognized precursor of end-stage joint failure [1,2,3]. Globally, knee osteoarthritis affects an estimated 365 million individuals aged over 40 years and constitutes one of the leading causes of disability, with prevalence rising in parallel with aging populations and escalating obesity rates [3,4]. Direct healthcare costs attributable to knee osteoarthritis in high-income countries exceed tens of billions of dollars annually [5].

Osteoarthritis is now understood as a heterogeneous whole-joint disorder involving articular cartilage, synovium, subchondral bone, menisci, ligaments, and periarticular tissues. Recent epidemiological and clinical evidence also indicates that the burden of knee osteoarthritis will continue to increase because of population aging, obesity, previous joint injury, and metabolic comorbidity [6,7,8,9].

The knee was selected as the focus of this review because it is the most frequently investigated joint in clinical cartilage-regeneration research and is exposed to high compressive, shear, and rotational loads during daily activity. These mechanical demands make knee cartilage repair particularly challenging and clinically relevant. Although MSC–hydrogel systems may also be applicable to other cartilaginous tissues, such as ankle, hip, shoulder, or temporomandibular cartilage, each anatomical site has distinct loading conditions, defect geometry, surgical accessibility, and synovial-fluid dynamics. Therefore, evidence from the knee cannot be directly generalized to other cartilage locations without dedicated clinical evaluation [3,4].

In addition, the biological performance of a hydrogel depends on joint-specific mechanical loading, lubrication, synovial-fluid turnover, defect geometry, and fixation conditions. Therefore, findings from knee studies should not automatically be extrapolated to the ankle, hip, shoulder, or temporomandibular joint [5,6].

Current surgical strategies span a wide spectrum. Microfracture is widely practiced owing to its technical simplicity and low cost, yet it reliably produces fibrocartilaginous repair tissue that is biomechanically inferior to native hyaline cartilage, and clinical benefits characteristically deteriorate beyond two years [6,7]. Autologous chondrocyte implantation (ACI) and its matrix-assisted variant generate more durable tissue but require a staged procedure and depend on the proliferative capacity of autologous chondrocytes [9,10,11]. Osteochondral transplantation is constrained by donor availability, size-matching requirements, and integration challenges, and none of these techniques addresses the diffuse cartilage loss of established osteoarthritis [12].

Despite continuous technical refinements, no currently available cartilage restoration technique consistently reproduces durable native hyaline cartilage across all defect types and patient populations, particularly in large lesions or osteoarthritic joints, underscoring the need for biologically augmented regenerative strategies [13,14,15].

MSC-based therapy has emerged as a compelling biological alternative. MSCs are multipotent stromal progenitors with chondrogenic differentiation capacity and a broad paracrine repertoire, secreting anti-inflammatory cytokines and trophic factors that attenuate synovitis and support residual chondrocytes [16,17]. Current evidence indicates that the therapeutic efficacy of MSCs is mediated predominantly through paracrine signaling rather than long-term engraftment or direct differentiation. Their secretome, extracellular vesicles, and immunomodulatory activity collectively regulate macrophage polarization, suppress inflammatory cytokine production, promote endogenous chondrogenesis, and support extracellular matrix remodeling [18,19,20]. MSC sources include bone marrow, adipose tissue, umbilical cord blood, and synovium, each conferring distinct proliferative and immunological characteristics [13,14]. Allogeneic UCB-MSCs combine high expansion capacity, hypo-immunogenicity, karyotypic stability, and freedom from donor-site morbidity, enabling GMP-compliant off-the-shelf products with national regulatory approval. Among currently available cell sources, umbilical cord-derived MSCs have attracted considerable attention because of their high proliferative potential, low immunogenicity, ease of expansion, and suitability for allogeneic off-the-shelf applications. Nevertheless, biological variability between tissue sources remains an important challenge for clinical standardization [21,22].

**Figure 1:**
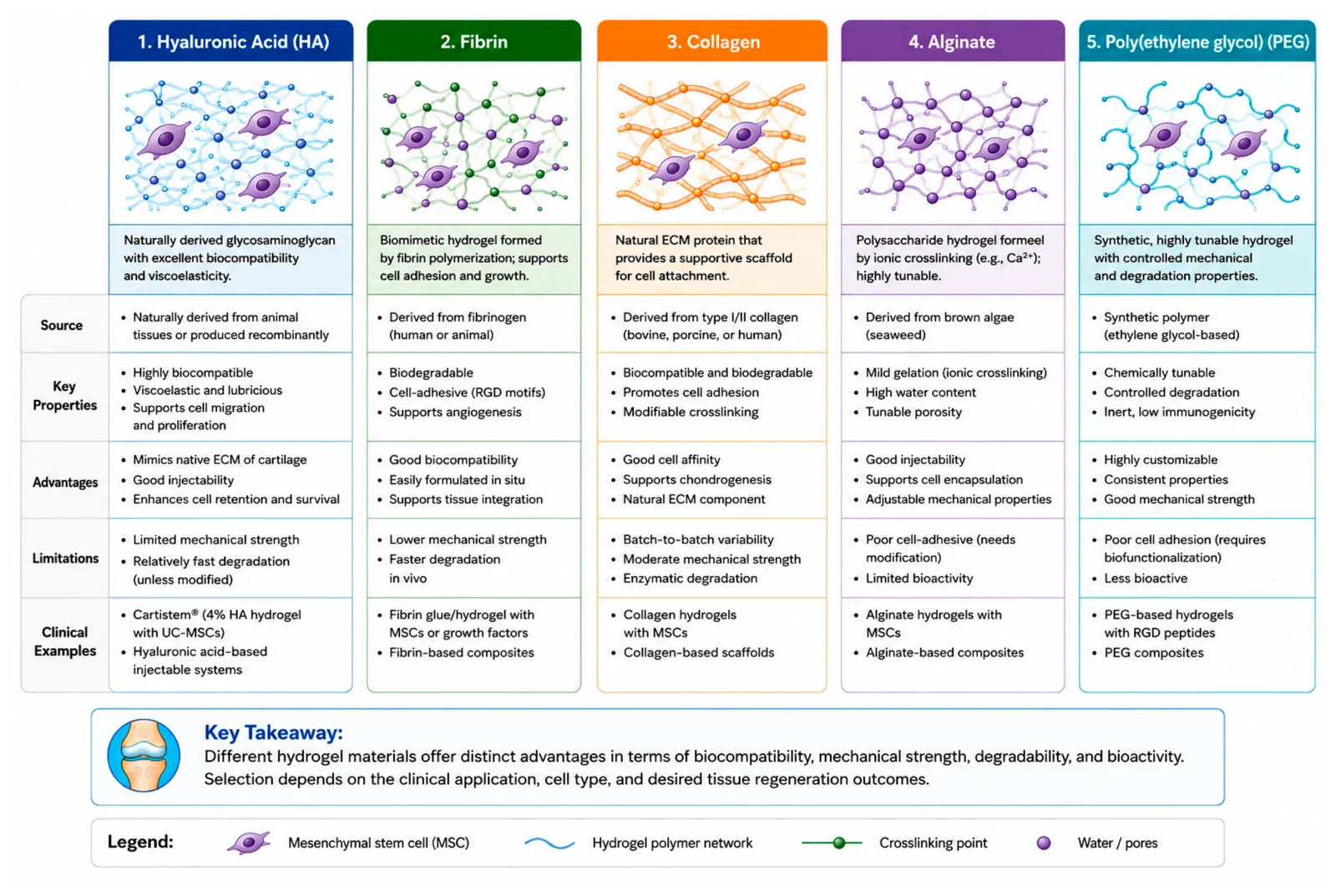
Types of hydrogels used for MSC delivery in cartilage repair. Natural hydrogels (hyaluronic acid, fibrin, collagen, alginate) and synthetic hydrogels (PEG) differ in biocompatibility, degradation kinetics, mechanical properties, and suitability for cartilage tissue engineering.

A central translational challenge is poor cell retention in the dynamic intra-articular environment: shear forces and synovial washout limit dwell time and spatial localization of injected cells [17,23]. Hydrogel-based delivery systems are three-dimensional, water-swollen polymer networks derived from hyaluronic acid, fibrin, collagen, alginate, or synthetic platforms that encapsulate MSCs within a biocompatible matrix, retaining cells at the defect site and enabling controlled growth factor release [19,20]. Beyond serving as passive carriers, modern hydrogels function as bioinstructive matrices capable of regulating cell behavior through their biochemical composition, mechanical stiffness, degradation kinetics, and spatial presentation of adhesion ligands. Advances in hydrogel engineering have enabled the development of injectable, self-healing, shear-thinning, adhesive, and stimuli-responsive systems specifically designed to improve cartilage regeneration and MSC function [21,22]. By addressing both structural and signaling determinants of regeneration, hydrogels are postulated to amplify efficacy beyond either component alone (Figure 1) [23,24].

Modern hydrogels may also function as instructive biomaterials by regulating matrix stiffness, porosity, ligand presentation, oxygen and nutrient diffusion, mechanotransduction, and the spatial release of bioactive molecules [25].

Natural hydrogels such as hyaluronic acid, fibrin, collagen, and alginate possess intrinsic biocompatibility and bioactivity but exhibit variable mechanical strength and degradation rates. Synthetic hydrogels such as polyethylene glycol (PEG) offer greater tunability and mechanical control but generally lack inherent biological signaling. These differences may influence MSC retention, differentiation, and long-term cartilage repair outcomes.

Hydrogels were selected as the focus of this review because they provide several advantages over many solid or semi-solid biopolymer scaffolds [25]. Their high-water content, extracellular matrix-like architecture, injectability or mouldability, and tunable degradation behavior allow them to support cell viability while adapting to irregular cartilage defects [21,25,26,27]. Compared with rigid membranes, sponges, or non-hydrated polymer scaffolds, hydrogels can more closely reproduce the hydrated microenvironment of native cartilage and can facilitate homogeneous MSC distribution within the defect. Nevertheless, hydrogels are not intrinsically superior in all contexts: their mechanical weakness, rapid degradation, or limited bioactivity may reduce performance unless their chemistry and crosslinking are optimized for the target joint environment [25,26,27].

More broadly, MSC–hydrogel systems belong to the expanding field of advanced biomaterials and composite technologies designed to improve human health through biologically active, minimally invasive, and potentially scalable therapeutic platforms. Recent developments in green nanotechnology, natural biomaterial composites, nanocomposite-enabled sensors, and 3D-printed bioactive scaffolds show that material design can support regenerative medicine, drug delivery, diagnostics, environmental remediation, clean-energy technologies, and sustainable manufacturing. This broader materials-science context is relevant because future cartilage-regeneration products should ideally combine biological efficacy, clinical safety, manufacturability, cost-effectiveness, and environmental responsibility [27,28,29,30].

Despite rapid preclinical progress [21,25,26,27,28,29,30,31,32,33,34,35,36,37,38,39,40,41], the clinical evidence for MSC–hydrogel composite therapy remains fragmented and without rigorous synthesis. The objectives of this review were to evaluate the safety and efficacy of MSC–hydrogel composites for knee cartilage defects and osteoarthritis in adults, to characterize effects across outcome domains, to examine the influence of hydrogel type and MSC source, and to assess the certainty of evidence using GRADE. To our knowledge, this is the most comprehensive and up-to-date systematic review on this topic, following PRISMA 2020 guidelines [42] and including formal risk-of-bias and GRADE assessments.

The contribution of this review is significant. First, it considers true MSC–hydrogel composite implantation evidence with contextual studies evaluating either MSCs without hydrogel or hydrogel without MSCs. Second, it maps clinical effects across cartilage repair, pain, function, quality of life, and safety domains while considering MSC source, hydrogel composition, and delivery route. Third, it links interpretation of efficacy to formal risk of bias and GRADE certainty assessments, thereby identifying the gap between promising regenerative technologies and the level of evidence required for international clinical translation.

## 2. Methods

This systematic review was conducted according to the Cochrane Handbook for Systematic Reviews of Interventions. The study is presented according to PRISMA 2020 guidelines [42].

### 2.1. Protocol Registration and Reporting Guidelines

This systematic review was prospectively registered in PROSPERO (CRD420261393525) and conducted in accordance with the Preferred Reporting Items for Systematic Reviews and Meta-Analyses (PRISMA 2020) Statement. Reporting of the narrative synthesis followed the Synthesis Without Meta-analysis (SWiM) guideline. A completed PRISMA 2020 checklist is provided as Supplementary Materials.

### 2.2. Eligibility Criteria

Eligible study designs were RCTs, controlled clinical trials, and prospective or retrospective cohort studies with ≥5 participants per arm. No restriction was placed on date, language, MSC source, or hydrogel type. Studies were excluded if conducted exclusively in animals or in vitro, or if the hydrogel functioned solely as an acellular scaffold in the clinical arm. Case series and case reports were excluded.

Studies that did not fully satisfy the intervention criterion of simultaneous MSC and structured hydrogel delivery were not included in the primary MSC–hydrogel synthesis. However, clinically relevant studies evaluating either hydrogel-based therapy without MSC administration or MSC therapy without a structured hydrogel carrier were eligible for separate contextual analysis when they helped interpret the independent contribution of the hydrogel scaffold or the MSC component. These studies were clearly labeled as contextual comparators and were not treated as direct evidence for MSC–hydrogel composite efficacy. The eligibility criteria based on the PICOS framework are summarized in Table 1.

### 2.3. Literature Search

The search was conducted in April–May 2026 across Embase, PubMed/MEDLINE, Cochrane CENTRAL, Scopus, and Web of Science, without date, language, or design filters. Three concept terms: mesenchymal stem cells, hydrogel, and knee cartilage were applied using database-specific controlled vocabulary and free text. Supplementary searches included reference list hand-searching, forward citation tracking via Scopus and Google Scholar, ClinicalTrials.gov registry search, and contact with trial authors for unpublished data. Detailed database-specific search strategies are provided in Supplementary Table S1.

### 2.4. Study Selection

**Figure 2:**
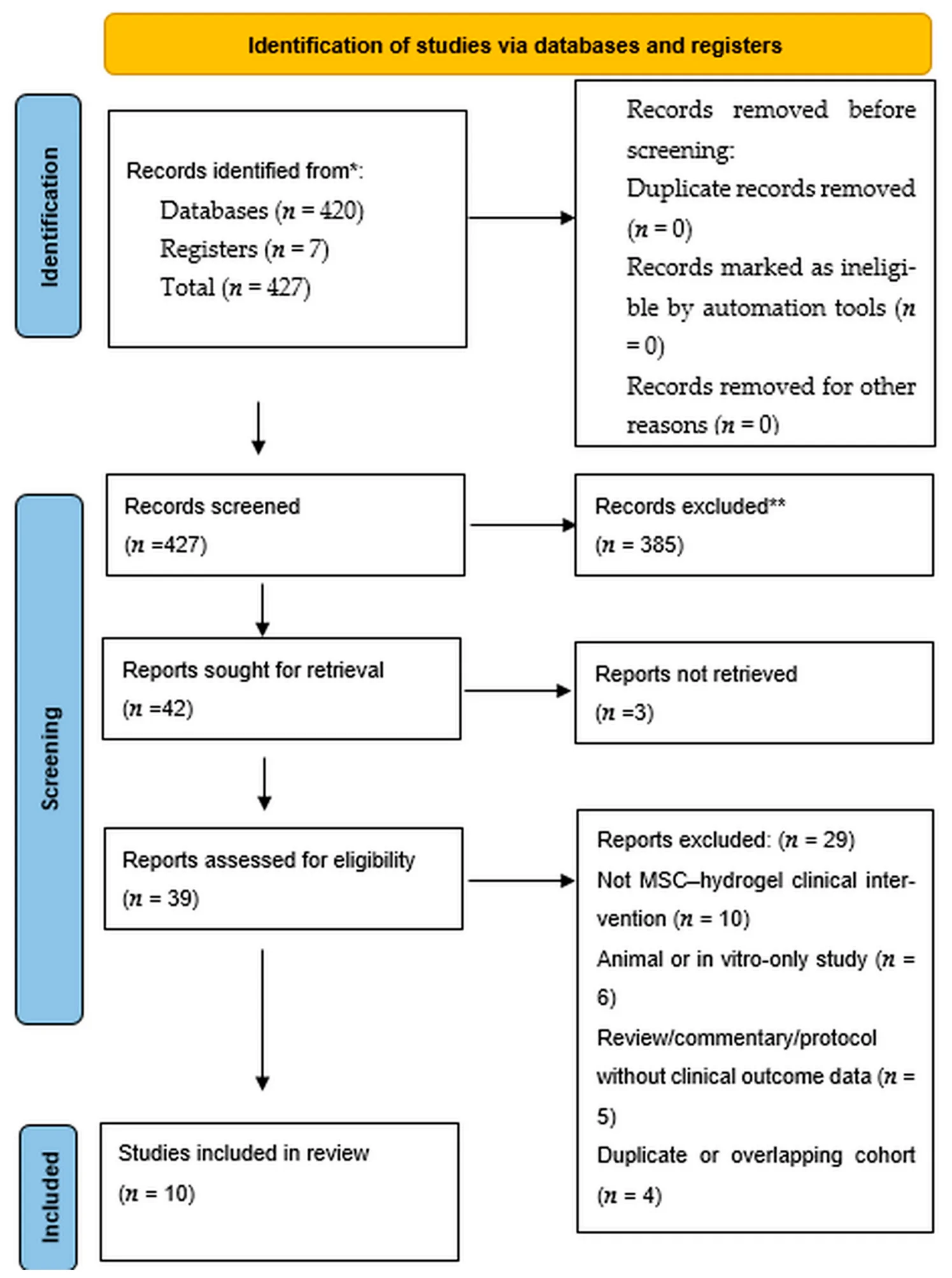
PRISMA 2020 flow diagram showing study identification, screening, eligibility assessment, and inclusion process. * Records were identified from databases and registers. ** Records were excluded manually by the reviewers during title and abstract screening; no automation tools were used for record exclusion.

All records were imported into Covidence systematic review software (Veritas Health Innovation, Melbourne, Australia; no version number assigned, https://www.covidence.org/, accessed on 1 July 2026) for automated deduplication. Two independent reviewers screened all titles and abstracts and then performed full-text review of eligible records. Interrater agreement was quantified using Cohen’s kappa (threshold: κ ≥ 0.70). Disagreements were resolved by discussion; unresolved disagreements were arbitrated by a third reviewer. The full selection process is presented in the PRISMA 2020 flow diagram (Figure 2).

### 2.5. Data Extraction

Data were extracted independently by two reviewers using a standardized Covidence form. Extracted variables included study design and registration status, patient demographics, MSC and hydrogel characteristics, delivery procedure, comparator, follow-up duration, pain and functional outcomes, quality-of-life measures, and adverse events. Discrepancies were resolved by discussion or by consulting a third reviewer.

### 2.6. Risk of Bias and Methodological Quality Assessment

RCTs were assessed using the Cochrane Risk of Bias tool, version 2 (RoB 2). Non-randomized studies were assessed using the Risk of Bias in Non-randomized Studies of Interventions (ROBINS-I) tool. Three independent reviewers performed assessments; disagreements were resolved by consensus. Interrater reliability was κ = 0.78.

### 2.7. Synthesis of Results

Given the substantial clinical and methodological heterogeneity across studies, including differences in MSC sources, hydrogel formulations, delivery methods, patient populations, outcome measures, and follow-up duration, a structured narrative synthesis was performed according to the framework proposed by Popay et al. [43] and reported in accordance with SWiM recommendations.

The synthesis followed four stages. First, a preliminary synthesis was developed by grouping studies according to intervention characteristics, including MSC source, hydrogel type, and delivery approach. Second, relationships within and between studies were explored by comparing outcomes across cartilage repair, pain, function, quality of life, and safety domains. Third, sources of heterogeneity were examined, including defect characteristics, osteoarthritis severity, hydrogel composition, and study design. Fourth, the robustness of the synthesis was assessed through formal risk of bias evaluation using RoB 2 and ROBINS-I and through certainty-of-evidence assessment using the GRADE framework.

To minimize subjective interpretation, data extraction, study selection, and risk of bias assessments were performed independently by multiple reviewers with consensus resolution of disagreements. Because substantial heterogeneity precluded quantitative pooling, findings were synthesized narratively and organized according to predefined outcome domains.

A supplementary random-effects meta-analysis (DerSimonian–Laird) was pre-specified as a contingency for outcomes reported by ≥3 studies using the same instrument at comparable timepoints; however, this criterion was not met, and therefore no quantitative meta-analysis was performed.

## 3. Results

### 3.1. Search Results and Study Selection

Database and registry searches retrieved 427 records after automated deduplication. Following title and abstract screening, 385 records were excluded, and 42 reports were sought for full-text retrieval. Three reports could not be retrieved. Of 39 full-text reports assessed for eligibility, 29 were excluded: 10 did not evaluate an MSC–hydrogel clinical intervention, six were animal or in vitro-only studies, five were reviews, commentaries, or protocols without clinical outcome data, four were duplicate or overlapping cohort reports, and four had insufficient sample size. Ten studies were included in the final narrative synthesis [44,45,46,47,48,49,50,51,52,53].

The study selection process is illustrated in a PRISMA 2020 flow diagram (Figure 2), including identification, screening, retrieval, eligibility assessment, and final study inclusion stages.

Of these ten studies, eight fulfilled the primary MSC–hydrogel implantation criterion. Two studies required a methodological clarification regarding their relationship to the PICOS criterion. Pipino et al. [45] used an acellular PG/GC thermogelling hydrogel as a microfracture augment in the clinical arm, whereas MSC encapsulation was evaluated only in a parallel in vitro experiment. Tong et al. [46] delivered umbilical cord-derived MSCs by intra-articular injection in a saline vehicle without a structured hydrogel scaffold. These two studies were retained as contextual comparator evidence to help interpret the contribution of the hydrogel component, but they were analyzed separately and were not treated as direct MSC–hydrogel implantation evidence. One ongoing Phase III RCT (NCT07339111) was identified through registry search; because no results had been posted at the data lock date, it was excluded from outcome synthesis.

### 3.2. Study Characteristics

Ten studies involving 521 patients were included [44,45,46,47,48,49,50,51,52,53]. Eight studies fulfilled the predefined eligibility criteria for MSC–hydrogel composite interventions and constituted the primary evidence synthesis [44,47,48,49,50,51,52,53]. These studies comprised one Phase I/II clinical trial, one randomized controlled trial, two prospective cohort studies, and four retrospective cohort studies. Two additional studies (Pipino et al., 2019 [45] and Tong et al., 2026 [46]) were retained as contextual comparators because they did not simultaneously evaluate both MSCs and a structured hydrogel carrier [45,46]. Sample sizes ranged from 15 to 128 participants. Mean patient age ranged from 44.2 years (Toktarov et al. [50]) to 58.7 years (Park et al. [44]). Seven studies enrolled patients with medial femoral condyle defects in the context of medial compartment osteoarthritis (predominantly KL grade II–III). Defect sizes, where reported, ranged from 4.5 to 7.8 cm^2^, predominantly exceeding the threshold above which conventional marrow stimulation is considered suboptimal. Full study-level characteristics are presented in Table 2.

Detailed intervention characteristics, including MSC source, hydrogel composition, cell dose, delivery method, defect size, and follow-up duration, are summarized in Supplementary Table S2.

### 3.3. MSC Sources and Hydrogel Carriers

Seven studies used allogeneic UCB-MSCs co-delivered in 4% lyophilized hyaluronic acid hydrogel (Cartistem^®^, Medipost, Seoul, Republic of Korea; 7.5 × 10^6^ cells/vial) [44,47,48,49,51,52,53]. One evaluated allogeneic umbilical cord-derived MSCs by intra-articular injection without a structured hydrogel [46]. One used autologous synovium-derived MSCs in a heparin-conjugated fibrin hydrogel supplemented with TGF-β1 (1 µg) and BMP-4 (1 µg) [50]. One used an acellular PG/GC thermogelling hydrogel as a microfracture augment, with MSCs evaluated only in vitro [45]. Cell doses ranged from 1.15 to 2.0 × 10^7^ cells. Two contextual comparator studies were analyzed separately. Pipino et al. [45] investigated an acellular PG/GC thermogelling hydrogel used as an adjunct to microfracture, whereas Tong et al. [46] evaluated intra-articular administration of UC-MSCs without a structured hydrogel scaffold. These studies were retained for contextual interpretation but were excluded from the primary MSC–hydrogel evidence synthesis [45,46].

### 3.4. Risk of Bias

Risk of bias assessments are presented in Table 3. The single Phase II RCT (Tong et al., 2026 [46]) was rated as having some concerns under RoB 2, primarily due to insufficient description of allocation concealment and the post hoc nature of the synovitis subgroup analyses that generated the study’s most salient positive findings. Among non-randomized studies, Lee et al., 2025 [52] was the only study rated at moderate overall risk; the remaining eight were rated serious (six studies) or critical (Song et al., 2020 [47]). Song et al., 2020 [47] was rated critical because the absence of a control group, concurrent microfracture in 75% of patients, and lack of confounder adjustment collectively preclude attribution of observed improvements to hUCB-MSC implantation. Low RoB: 0 (RCT with some concerns); Moderate: 3/10; Serious: 6/10; Critical: 1/10. Interrater reliability: κ = 0.78. The characteristics of MSC sources, hydrogel formulations, delivery methods, and study-specific interventions are summarized in Table 3.

### 3.5. Results by Outcome

Outcome analyses were primarily based on the eight studies evaluating true MSC–hydrogel composite interventions. Contextual comparator studies were considered separately to explore the independent contribution of hydrogel scaffolds and MSC administration.

#### 3.5.1. Cartilage Repair Outcomes

The most comprehensive cartilage repair data derive from Park et al., 2017 [44]. Arthroscopic evaluation at 12 weeks demonstrated maturing repair tissue in all evaluable participants. Histological analysis at one year (***n*** = 2) revealed tissue with strong type II collagen, Safranin O, and Masson’s trichrome positivity, closely resembling native hyaline cartilage. dGEMRIC MRI at three years (***n*** = 5) demonstrated mean relative ΔR1 = 1.44, indicating glycosaminoglycan content comparable to native cartilage, with clinical improvements maintained without significant deterioration over seven years and no arthroplasty conversions.

Among retrospective cohorts:

- Jung et al., 2025 [53] demonstrated a significant location-dependent gradient: anterior-to-central defects achieved mean MOCART 2.0 of 55.0 ± 12.3 versus 40.4 ± 9.2 for posteriorly extending defects (*p* = 0.001). A defect size cut-off of 6.3 cm^2^ (AUC 0.905) predicted unsatisfactory regeneration (MOCART < 60) with sensitivity 0.786 and specificity 0.933.
- Lee et al., 2025 [52] found no significant difference in MOCART 2.0 by meniscal status (56.8 ± 10.5 vs. 54.1 ± 8.8; *p* = 0.347), but preoperative meniscal extrusion negatively correlated with MOCART 2.0 (r = −0.24, *p* = 0.029) and subchondral changes (r = −0.29, *p* = 0.017).
- Suh et al., 2021 [48] demonstrated greater joint space width increment in the hUCB-MSC group versus microfracture (0.6 ± 0.9 mm vs. 0.1 ± 1.3 mm; *p* = 0.036).
- Toktarov et al., 2022 [50] reported MOCART improvement in 73.3% of patients and complete graft integration in 73.3% at six months.
- Kim et al., 2023 [51] found ICRS grade I/II in 64% of hUCB-MSC patients versus 44% of SVF patients (*p* = 0.170), with ICRS grade significantly correlated with all KOOS subscores and IKDC in both groups (all *p* < 0.05).
- Tong et al., 2026 [46] evaluating injection without hydrogel scaffolding found no significant change in cartilage defect size or bone marrow lesions at six months, but demonstrated significant synovitis reduction versus placebo and HA (*p* = 0.003), with complete synovitis resolution in 61.5% of UC-MSC-treated patients versus 18.2% (placebo) and 30.0% (HA).

#### 3.5.2. Pain Outcomes

Pain assessed by VAS was reported in seven studies and consistently improved across surgical implantation studies. Park et al., 2017 [44] demonstrated VAS improvement from 49.1 to 19.3 at 24 weeks (*p* = 0.018), sustained without significant deterioration over seven years. Song et al., 2020 [47] showed progressive reduction from 7.0 ± 1.6 at baseline to 2.5 ± 1.7 at one year and 2.0 ± 2.1 at final follow-up (all *p* < 0.001); medial femoral condyle lesions were associated with worse VAS than trochlear lesions (*p* < 0.05). Toktarov et al., 2022 [50] documented VAS improvement from 67.54 ± 13.73 to 14.74 ± 17.20 at six months (*p* < 0.001). Jung et al., 2025 [53] found significant VAS improvement in both defect-location groups (all *p* < 0.001) without significant between-group difference. In Tong et al., 2026 [46], no significant VAS difference was observed in the overall intention-to-treat population; however, in the KOA-plus-synovitis subgroup with moderate-to-severe pain (VAS ≥ 4), UC-MSC injection produced significantly superior pain reduction versus placebo and HA at 24 weeks (*p* = 0.011).

#### 3.5.3. Functional Outcomes

IKDC subjective score, reported in five studies, consistently improved following MSC–hydrogel implantation. Park et al., 2017 [44]: 39.1 → 63.2 at 24 weeks (*p* = 0.018), stable over seven years. Song et al., 2020 [47]: 32.5 → 55.8 at one year → 61.2 at final follow-up (all *p* < 0.001; final follow-up scores significantly better than one-year scores, *p* < 0.001, indicating continued maturation). Suh et al., 2021 [48]: IKDC 69.0 ± 7.7 (MSC) vs. 62.1 ± 12.9 (microfracture) at 18 months (*p* = 0.002); HSS and Lysholm did not differ significantly. Kim et al., 2023 [51]: IKDC and all KOOS subscores improved significantly in both SVF and hUCB-MSC groups without significant between-group differences at any timepoint (all *p* > 0.05).

WOMAC was reported in five studies. Pipino et al., 2019 [45]—noting its eligibility caveat—reported a total WOMAC score of 2.9 ± 5.9 (hydrogel + MFx) versus 48.3 ± 13.3 (MFx alone) at 24 months (*p* < 0.0001), representing a 95.1% versus 14.5% reduction from baseline; the magnitude of this effect must be interpreted in the context of its serious risk of bias, particularly the baseline pain imbalance and sequential allocation design. Toktarov et al., 2022 [50] documented WOMAC-C function improvement from 23.58 to 6.45 at six months (*p* < 0.001), alongside comprehensive KOOS improvement across all subscores. In Tong et al., 2026 [46], WOMAC improvement reached significance in the synovitis subgroup at 24 weeks (*p* = 0.014) but not in the overall population; EQ-5D similarly improved significantly in the synovitis subgroup (*p* = 0.049). Lee et al., 2025 [52] and Jung et al., 2025 [53] found significant within-group WOMAC improvements from baseline (*p* < 0.001) without significant between-group differences in their comparative analyses.

#### 3.5.4. Safety

The safety profile was uniformly favorable across all ten included studies. No study recorded osteogenesis, tumorigenesis, ectopic bone formation, septic arthritis, permanent disability, or treatment-related death. Park et al., 2017 [44] observed five mild-to-moderate TEAEs across seven participants over seven years; only one (antithyroglobulin elevation, spontaneously resolving) was classified as treatment-related. Tong et al., 2026 [46] reported adverse events in 29.4% of UC-MSC recipients versus 18.8% (HA) and 0% (placebo), all mild-to-moderate and without between-group difference (*p* = 0.091). No serious adverse events were reported in any remaining study. Adverse events were inconsistently documented across studies, so the evidence is insufficient to draw definitive conclusions about the full safety profile; minor events such as transient joint pain and injection-site discomfort may be underreported [54].

### 3.6. GRADE Assessment

GRADE certainty assessments per outcome domain are presented in Table 4. Evidence certainty was very low for cartilage repair and WOMAC outcomes, low for pain (VAS), function (IKDC), and safety. The predominant downgrading reasons were serious or critical risk of bias across the non-randomized evidence base, serious imprecision from small sample sizes, indirectness from concentration of evidence around a single regulatory-approved product in a single geographic region, and inconsistency in outcome instrument and follow-up duration. A summary of the clinical outcomes and evidence synthesis is presented in Table 4.

## 4. Discussion

This systematic review synthesizes evidence from ten studies (521 patients) on MSC–hydrogel composite systems for knee cartilage repair and osteoarthritis. All included studies reported improvements in cartilage repair, pain, and/or function following MSC–hydrogel treatment, with no major adverse events documented across any study. However, the evidence base is dominated by small retrospective cohorts, most using a single regulatory-approved product in a single geographic setting, limiting the certainty of conclusions (GRADE: low to very low).

Despite these limitations, three findings are of clinical significance. First, surgical implantation of MSC–hydrogel composites, which were predominantly the allogeneic UCB-MSC/4% HA formulation Cartistem^®^, produced histologically verifiable hyaline-like cartilage at one year, with dGEMRIC-confirmed glycosaminoglycan content approaching native values at three years and clinical stability over seven years without arthroplasty conversion [44]. This durability profile substantially exceeds the one-to-two-year clinical deterioration that characterizes microfracture-derived fibrocartilage [9,10] and represents the most compelling mechanistic differentiation between these approaches. These observations are consistent with recent evidence indicating that the long-term limitations of marrow stimulation are primarily attributable to fibrocartilage formation, inferior biomechanical properties, and progressive structural deterioration under physiological joint loading. In contrast, cell-based regenerative strategies seek to restore a more hyaline-like extracellular matrix and improve long-term tissue durability [9,13].

Second, cartilage repair quality, as measured by ICRS grade, significantly correlated with functional outcomes (IKDC, KOOS) in the only direct comparative study between hUCB-MSC and autologous SVF [51], confirming that structural regeneration mediates functional recovery rather than merely reflecting parallel biological processes. Third, intra-articular injection without a hydrogel scaffold [46] produced anti-inflammatory effects specifically significant for synovitis reduction but no detectable structural cartilage regeneration at six months, and clinical benefits were confined to patients with concurrent synovitis and moderate-to-severe baseline pain. This contrast suggests that the hydrogel scaffold provides essential added value beyond cell delivery alone, by retaining cells at the defect site and providing a three-dimensional chondrogenic microenvironment [27,30,55].

This apparent dissociation between structural cartilage repair and patient-reported clinical outcomes has been consistently reported in cartilage restoration research. Although MRI- and arthroscopy-based assessments frequently demonstrate progressive maturation of repair tissue, improvements in pain and function are additionally influenced by age, baseline osteoarthritis severity, limb alignment, meniscal integrity, inflammatory status, rehabilitation protocols, and patient expectations. Therefore, structural regeneration should be interpreted as a complementary endpoint rather than a direct surrogate for clinical success [56,57].

The available clinical evidence indirectly suggests that surgical implantation may provide superior structural regeneration compared with intra-articular injection. Studies using implanted MSC–hydrogel constructs demonstrated consistent improvements in cartilage repair outcomes, including MOCART, ICRS grading, and histological findings, whereas the only injection-based study showed anti-inflammatory benefits without detectable structural cartilage regeneration. However, direct comparative trials are lacking, and firm conclusions cannot yet be drawn [58]. A comparative overview of MSC–hydrogel systems and established cartilage repair strategies is presented in Table 5.

### 4.1. Biological Mechanisms of MSC–Hydrogel-Mediated Cartilage Regeneration

**Figure 3:**
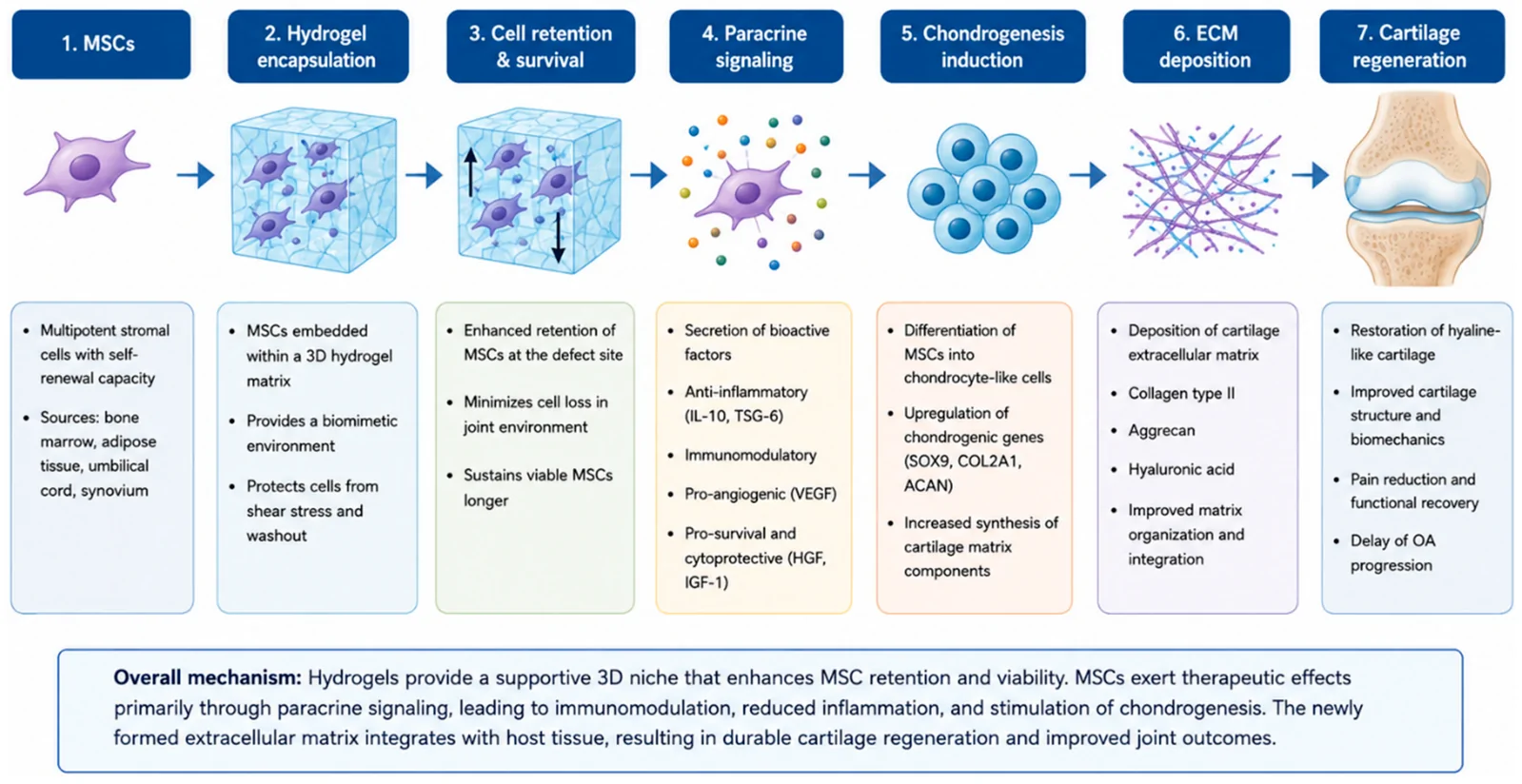
Proposed biological mechanisms of MSC–hydrogel-mediated cartilage repair. Hydrogels improve MSC retention and survival within the defect site, enabling sustained paracrine signaling, chondrogenic differentiation, extracellular matrix deposition, and progressive cartilage regeneration. **Color note:** Different colors are used solely to visually distinguish the sequential stages of the proposed mechanism and do not represent different cell types, hydrogel materials, signaling pathways, or levels of biological activity.

Hydrogels provide a three-dimensional microenvironment that enhances MSC retention, viability, and local bioactivity within the cartilage defect. Encapsulated MSCs exert therapeutic effects through paracrine signaling, including secretion of anti-inflammatory cytokines and trophic growth factors [59]. These signals promote chondrogenic differentiation, extracellular matrix synthesis, and modulation of the inflammatory joint environment. Progressive deposition of collagen type II, aggrecan, and other cartilage-specific matrix components facilitates integration of repair tissue and restoration of cartilage structure and function (Figure 3). Recent advances further indicate that the therapeutic activity of MSC–hydrogel constructs is governed not only by cell survival but also by reciprocal interactions between encapsulated MSCs and the surrounding biomaterial [60]. Hydrogel stiffness, pore architecture, degradation kinetics, and biochemical functionalization regulate mechanotransduction pathways, influence MSC lineage commitment, and shape the composition of the newly synthesized extracellular matrix [61]. Simultaneously, the hydrogel protects encapsulated cells from mechanical stress and rapid intra-articular clearance, thereby prolonging local paracrine activity throughout the repair process [21,22,25,26].

### 4.2. Hydrogel Degradation Kinetics and Cartilage Regeneration

Hydrogel degradation kinetics represent an important determinant of successful cartilage regeneration. An ideal hydrogel scaffold should provide sufficient mechanical support and cell retention during the early stages of repair while gradually degrading as newly formed extracellular matrix develops. This temporal synchronization allows progressive transfer of mechanical load from the biomaterial to the regenerating tissue [62].

Excessively rapid degradation may lead to premature loss of structural support, reduced cell retention, and impaired tissue maturation. Conversely, overly slow degradation may limit matrix remodeling, interfere with host tissue integration, and restrict nutrient diffusion. Therefore, matching degradation kinetics to the rate of cartilage matrix formation is considered a key design principle for next-generation MSC–hydrogel systems. Recent advances in biomaterial engineering have enabled the development of smart hydrogels with programmable degradation profiles that respond to enzymatic activity, pH changes, or mechanical loading within the joint environment. Such systems provide temporary mechanical support during the early stages of cartilage repair while gradually transferring load-bearing function to the newly synthesized extracellular matrix [63]. This synchronized remodeling process promotes tissue integration, facilitates matrix maturation, and may ultimately improve the long-term durability of cartilage regeneration [21,22,25,26].

The predominance of hyaluronic acid-based hydrogels in the current clinical literature may partly reflect their favorable biodegradation profile, biocompatibility, and ability to support chondrogenic differentiation. However, comparative clinical studies directly evaluating the relationship between hydrogel degradation rate and long-term cartilage repair outcomes remain limited and should be considered a priority for future research.

Several modifiers of treatment response were identified. Jung et al., 2025 [53] demonstrated that defect location within the medial femoral condyle significantly determines regeneration quality, with anterior-to-central defects outperforming posteriorly extending defects (MOCART 2.0: 55.0 vs. 40.4; *p* = 0.001), and a validated defect size threshold of 6.3 cm^2^ above which unsatisfactory outcomes occur with high specificity (0.933). These findings define practical patient selection parameters. Lee et al., 2025 [52] identified preoperative meniscal extrusion as a negative predictor of subchondral integrity after treatment. Tong et al., 2026 [46] identified synovitis-positive patients as the specific phenotype most likely to respond to intra-articular injection. Together, these data indicate that stratification by defect size, location, and synovitis status is essential for optimizing patient selection and for enriching future trial populations.

Beyond patient selection, further progress will depend on the development of injectable hydrogels with improved bioadhesion, controlled degradation, enhanced integration with native cartilage, and the capacity to deliver cells or bioactive molecules in a spatially controlled manner. In addition, successful clinical translation will require optimization of manufacturing processes, regulatory approval pathways, and long-term safety evaluation [55].

The growth factor-supplemented heparin-conjugated fibrin hydrogel evaluated by Toktarov et al., 2022 [50] is the only example in this corpus of a purpose-designed controlled-release scaffold. MOCART improvement in 73.3% and complete graft integration in 73.3% at six months suggest a performance profile comparable to Cartistem^®^ cohorts at equivalent timepoints, despite using autologous rather than allogeneic cells. This represents an important proof of concept for next-generation MSC–hydrogel composites incorporating sustained chondroinductive signaling.

There is no systematic review evaluating the efficacy of MSC–hydrogel implantation for knee cartilage defects. Moreover, the present analysis advances the field through stricter inclusion criteria, an updated evidence base to May 2026, formal ROBINS-I and RoB 2 risk of bias assessment, and GRADE evaluation of certainty across all outcome domains.

### 4.3. Hydrogel Chemistry, Encapsulation, and Delivery Approach

From a chemical and procedural perspective, the included hydrogels differ substantially. Hyaluronic-acid hydrogels provide a naturally cartilage-associated matrix and are commonly used because of their biocompatibility, biodegradability, and clinical familiarity [64]. Fibrin-based hydrogels provide a provisional matrix that can support cell adhesion and tissue integration, while heparin-conjugated fibrin systems may additionally retain and release chondroinductive growth factors such as TGF-β1 and BMP-4. Synthetic or semi-synthetic systems, including PEG-based or thermogelling composites, offer greater control over crosslinking, stiffness, and degradation but may require additional biofunctionalization to support cell adhesion and chondrogenesis [27,30,50,65].

Recent advances have further expanded hydrogel engineering toward bioinstructive systems capable of responding to environmental stimuli and dynamically regulating cell behavior, thereby improving cartilage-specific matrix formation and tissue integration [58].

Encapsulation generally involves mixing MSCs with a hydrogel precursor or carrier before gelation or implantation, allowing cells to be distributed within a three-dimensional scaffold. Surgical implantation may provide better defect filling, spatial localization, and mechanical stability, whereas intra-articular injection is less invasive but may be more vulnerable to synovial dilution, shear forces, and poor cell retention. Based on the current evidence, implanted MSC–hydrogel constructs appear more consistently associated with structural cartilage repair, while injection-based MSC therapy without hydrogel showed mainly anti-inflammatory effects. However, direct trials comparing injection and implantation approaches are still lacking.

### 4.4. Clinical Implications

The findings of this review suggest that MSC–hydrogel composite systems may represent a promising treatment option for selected patients with focal cartilage defects and early-to-moderate knee osteoarthritis who are not ideal candidates for conventional cartilage restoration procedures. The available evidence indicates potential benefits in cartilage repair, pain reduction, and functional improvement, particularly when MSCs are delivered within a structured hydrogel scaffold.

From a practical perspective, hydrogel-assisted MSC delivery may improve cell retention within the defect site and facilitate localized tissue regeneration. Although current evidence remains insufficient to support routine clinical adoption, these technologies may serve as an adjunct or alternative to marrow stimulation techniques in carefully selected patients.

Despite encouraging findings from recent comparative studies, the available evidence remains heterogeneous, precluding firm recommendations for routine clinical implementation. Gaissmaier et al. [65] demonstrated that hydrogel-based matrix-associated autologous chondrocyte implantation achieved greater improvements in pain, symptoms, sports function, and quality of life than microfracture at 24-month follow-up, further supporting the clinical potential of hydrogel-assisted cartilage repair strategies.

Niemeyer et al. [66] reported the 5-year results from a prospective multicenter phase III trial have demonstrated sustained clinical improvement and a favorable safety profile following hydrogel-based autologous chondrocyte implantation for large knee cartilage defects, supporting the long-term durability of this regenerative strategy.

Mumme et al. [67] demonstrated that enhanced maturation of tissue-engineered cartilage was associated with superior clinical and compositional outcomes, further highlighting the importance of scaffold design and graft quality for durable cartilage regeneration.

Collectively, these recent clinical and translational findings indicate that the future of cartilage regeneration will depend not only on the choice of cell source but also on the development of biomimetic scaffolds capable of recreating the native cartilage microenvironment and promoting long-term tissue maturation. Recent advances in hydrogel engineering, including extracellular matrix-based biomaterials, dynamic viscoelastic hydrogels, bioactive molecule-loaded systems, and emerging multifunctional immunomodulatory scaffold platforms, are expected to further improve cell retention, chondrogenic differentiation, graft integration, and structural repair of articular cartilage [68,69,70,71,72].

An important limitation of the current evidence base is that seven of the eight included MSC–hydrogel studies evaluated a single commercial platform (Cartistem^®^), potentially limiting the generalizability of these findings to other hydrogel formulations, biomaterial compositions, and MSC products.

Beyond clinical validation, successful translation of MSC–hydrogel technologies into routine orthopedic practice will require standardized manufacturing protocols, Good Manufacturing Practice (GMP)-compliant production, reproducible biomaterial fabrication, scalable processing, and clear regulatory pathways for advanced therapy medicinal products. Addressing these translational challenges will be essential to ensure consistent product quality, regulatory approval, and broad clinical accessibility [56,57].

To facilitate broader clinical adoption, future research should prioritize large, multicenter randomized controlled trials comparing MSC–hydrogel composite implantation with microfracture, ACI/MACI, and osteochondral transplantation. Future studies should also stratify outcomes according to defect size, lesion location, and synovitis status. The ongoing Phase III Medipost trial (NCT07339111) comparing Cartistem^®^ with arthroscopic debridement is expected to provide important evidence regarding long-term efficacy. Furthermore, universal adoption of standardized outcome measures, including MOCART 2.0, IKDC, KOOS, and VAS, together with cost-effectiveness analyses, will be essential for future evidence synthesis and health-care decision-making.

## 5. Conclusions

Current clinical evidence suggests that MSC–hydrogel composite systems may improve cartilage repair, pain, and functional outcomes in patients with focal knee cartilage defects and knee osteoarthritis. The available studies generally report favorable safety findings and encouraging signals of structural cartilage regeneration, including histological evidence of hyaline-like repair tissue and sustained clinical improvements in some cohorts.

However, confidence in these findings remains limited because small single-center studies, retrospective designs, substantial risk of bias, and low-to-very-low certainty of evidence according to GRADE assessments dominate the evidence base. Furthermore, most available clinical data originate from a single hydrogel platform and a limited geographic region, reducing generalizability.

At present, MSC–hydrogel implantation should be regarded as a promising but still evolving regenerative strategy rather than an established standard of care. Future multicenter randomized controlled trials with standardized outcome reporting, longer follow-up, and direct comparisons with microfracture, ACI/MACI, and osteochondral transplantation are required to determine the true clinical value of these technologies.

Particular research priorities include optimization of hydrogel composition, degradation kinetics, MSC source selection, and patient stratification according to defect size, location, and inflammatory status. These advances may help define the role of MSC–hydrogel systems in next-generation cartilage regeneration.

Key translational challenges include standardizing MSC dose, hydrogel composition, degradation kinetics, implantation technique, imaging endpoints, adverse-event reporting, regulatory requirements, and cost-effectiveness evaluation. Addressing these challenges will be essential before MSC–hydrogel composites can move from promising regenerative technologies to broadly accepted clinical interventions.

## Figures and Tables

**Table 1:** Eligibility criteria (PICOS framework).

Code	Element	Criterion
P	Population	Adults (≥18 yrs) with focal chondral/osteochondral knee defects or knee osteoarthritis with documented cartilage damage
I	Intervention	MSCs from any human tissue source (bone marrow, adipose, umbilical cord blood, synovium) co-delivered within a hydrogel carrier (HA, fibrin, alginate, PEG, chitosan, collagen, or composite), administered by intra-articular injection or surgical implantation
C	Comparator	Any: microfracture, ACI/MACI, hyaluronic acid alone, PRP, MSCs without hydrogel, sham procedure, no treatment, or single-arm pre–post designs
O	Outcomes	Cartilage repair (primary): ICRS grade, MOCART score, MRI, histology. Secondary: pain (VAS), function (IKDC, WOMAC, KOOS), quality of life (EQ-5D), safety (adverse events)
S	Study design	RCTs, controlled clinical trials, prospective/retrospective cohort studies; ≥5 participants per arm; case reports excluded

**Table 2:** Summary of included studies.

Author, Year	Design	*N* (Total)	*N* Post-MSC	Mean Age (yr)	MSC Source	Hydrogel	Primary Outcome
Park et al., 2017 [44]	Phase I/II single-arm	7	7	58.7	Allogeneic UCB-MSC	4% HA hydrogel (Cartistem^®^)	ICRS repair at 12 wk; VAS; IKDC over 7 yr
Song et al., 2020 [47]	Retrospective series	128	128	56.5	Allogeneic UCB-MSC	4% HA hydrogel (Cartistem^®^)	VAS; WOMAC; IKDC at ≥2 yr
Suh et al., 2021 [48]	Retrospective case–control	100 knees	43 (MSC arm)	56.5	Allogeneic UCB-MSC	4% HA hydrogel (Cartistem^®^)	IKDC at 18 months; JSW
Lim et al., 2021 [49]	Multicenter RCT (5-yr FU)	NR	NR	NR	Allogeneic UCB-MSC	4% HA hydrogel (Cartistem^®^)	ICRS grade; VAS; IKDC over 5 yr
Toktarov et al., 2022 [50]	Prospective single-arm	15	15	44.2	Autologous Sy-MSC	Heparin-conjugated fibrin + TGF-β1/BMP-4	Safety; VAS; WOMAC; KOOS; MOCART at 6 months
Kim et al., 2023 [51]	Retrospective cohort (matched)	50	25 (hUCB-MSC)	56.4	Allogeneic UCB-MSC	4% HA hydrogel (Cartistem^®^)	IKDC; KOOS; ICRS grade at second-look (~12.6 months)
Lee et al., 2025 [52]	Retrospective cohort	47	47	57.2	Allogeneic UCB-MSC	4% HA hydrogel (Cartistem^®^)	IKDC; WOMAC; MOCART 2.0 at ≥2 yr
Jung et al., 2025 [53]	Retrospective cohort	43	43	56.1	Allogeneic UCB-MSC	4% HA hydrogel (Cartistem^®^)	MOCART 2.0 at 1 yr; VAS; IKDC; KOOS; WOMAC

*Cartistem^®^ is manufactured by MEDIPOST Co., Ltd. (Seongnam-si, Gyeonggi-do, Republic of Korea).*

**Table 3:** Risk of bias assessment by study.

Author, Year	Tool	Confounding/Randomisation	Blinding/Deviations	Missing Data	Outcome Measurement	Overall RoB
Park et al., 2017 [44]	ROBINS-I	Moderate (no comparator, ***n*** = 7)	Low (single-arm)	Low (all completed 7-yr FU)	Low (independent arthroscopist)	Moderate
Pipino et al., 2019 [45]	ROBINS-I	Serious (sequential allocation; baseline pain imbalance)	Serious (treatment packages differ between arms)	Moderate	Low (WOMAC validated)	Serious
Song et al., 2020 [47]	ROBINS-I	Critical (no control; 75% concurrent MFx; no confounder adjustment)	Critical	Moderate	Low	Critical
Suh et al., 2021 [48]	ROBINS-I	Serious (no preoperative clinical scores)	Moderate	Low	Low	Serious
Lim et al., 2021 [49]	RoB 2	Low (computer randomization)	High (open-label; different surgical procedures)	Low (ITT)	Low (blinded assessors)	Some concerns
Toktarov et al., 2022 [50]	ROBINS-I	Moderate (no control; OA patients only)	Low (single-arm)	Low (all 15 completed 6-month FU)	Moderate (MOCART rater NR)	Moderate
Kim et al., 2023 [51]	ROBINS-I	Serious (retrospective; different hydrogel carriers; indication bias)	Moderate	Moderate	Low	Serious
Tong et al., 2026 [46]	RoB 2	Low (double-blind placebo-controlled)	Low (double-blind)	Low	Some concerns (post hoc subgroup analyses drove main positive findings)	Some concerns
Lee et al., 2025 [52]	ROBINS-I	Moderate (single surgeon; balanced baselines; prospectively registered)	Low	Low	Low (MOCART 2.0 by radiologist)	Moderate
Jung et al., 2025 [53]	ROBINS-I	Serious (groups differed in defect size and kissing lesion prevalence; no adjustment)	Moderate	Low	Low	Serious

**Table 4:** Summary of findings—GRADE certainty of evidence.

Outcome (Studies; *N*)	Risk of Bias	Inconsistency	Indirectness	Imprecision	Publication Bias	Certainty	Effect and Interpretation
Cartilage repair: MOCART 2.0/ICRS grade (5 studies; ***n*** = 268)	Serious	Serious	Serious	Serious	Suspected	Very low	Consistently positive direction (MOCART 40–57 at 1 yr; hyaline-like histology in 1 biopsy study). Cannot reliably attribute to intervention given uncontrolled designs.
Pain: VAS (6 studies; ***n*** = 370)	Serious	Not serious	Serious	Serious	Suspected	Low	Significant within-group improvement in all surgical implantation studies. RCT significant in synovitis subgroup only. Whether effect exceeds sham or natural history cannot be determined.
Function: IKDC (5 studies; ***n*** = 333)	Serious	Not serious	Serious	Serious	Suspected	Low	Significant improvement across all studies (preoperative: ~33–42; postoperative: ~58–71).
Function: WOMAC (5 studies; ***n*** = 360)	Serious	Serious	Serious	Serious	Suspected	Very low	Consistent within-group improvement. Extreme difference in highest RoB study; RCT significant in synovitis subgroup only. Effect size cannot be reliably estimated.
Quality of life: EQ-5D (1 study; ***n*** = 17)	Some concerns	N/A	Serious	Very serious	Undetected	Very low	Significant in KOA + synovitis subgroup at 24 weeks (*p* = 0.049). Single RCT subgroup analysis; cannot generalize.
Safety: absence of serious AEs (9 studies; ***n*** = 506)	Serious	Not serious	Not serious	Serious	Unlikely	Low	No serious treatment-related AEs, osteogenesis, or tumorigenesis reported across any study over 6 months–7 years. Most consistent finding in this corpus.

Abbreviations: EQ-5D, EuroQol 5-Dimension questionnaire; KOA, knee osteoarthritis; RCT, randomized controlled trial; N/A, not applicable.

**Table 5:** Clinical comparison of MSC–hydrogel systems with other cartilage-repair strategies.

Approach	Main Advantage	Main Limitation	Relevance to Current Review
Microfracture	Simple, low-cost, and widely available	Produces fibrocartilage with limited durability, especially in larger defects	Most common comparator; MSC–hydrogel systems may offer improved structural repair, but certainty remains low
ACI/MACI	Can generate more durable repair tissue	Requires a staged procedure, cell expansion, and higher cost	Important benchmark for biologically enhanced cartilage repair
Osteochondral transplantation	Transfers mature osteochondral tissue	Limited by donor-site morbidity, graft availability, size matching, and integration challenges	Useful comparator for focal defects but less applicable to diffuse osteoarthritis
MSC injection without hydrogel	Minimally invasive and may reduce inflammation	Limited cell retention and weak evidence for structural regeneration	Contextual evidence suggests MSCs alone may improve synovitis but not cartilage repair
Acellular hydrogel augmentation	Provides scaffold support without cell therapy	Does not directly test MSC–hydrogel synergy	Useful contextual comparator for isolating scaffold-related effects
MSC–hydrogel composite implantation	Combines cell therapy with localized scaffold-based retention and a chondrogenic microenvironment	Evidence is mostly non-randomized, geographically concentrated, and product-specific	Main intervention of this review; promising but not yet established as standard care

## Data Availability

All data generated or analyzed during this study are included in this published article and its Supplementary Materials.

## Supplementary Materials

The following supporting information can be downloaded at: https://www.mdpi.com/article/10.3390/gels12080715/s1, Supplementary Checklist S1. PRISMA 2020 Checklist; Table S1: Complete database-specific search strategies used for study identification; Table S2: Technical Characteristics of Included MSC–Hydrogel Studies.

## Abbreviations

ACI, autologous chondrocyte implantation; AE, adverse event; BMP-4, bone morphogenetic protein-4; dGEMRIC, delayed gadolinium-enhanced magnetic resonance imaging of cartilage; ECM, extracellular matrix; GRADE, Grading of Recommendations Assessment, Development and Evaluation; HA, hyaluronic acid; hUCB-MSC, human umbilical cord blood-derived mesenchymal stem cell; ICRS, International Cartilage Repair Society; IKDC, International Knee Documentation Committee; KOOS, Knee injury and Osteoarthritis Outcome Score; MACI, matrix-assisted autologous chondrocyte implantation; MOCART, Magnetic Resonance Observation of Cartilage Repair Tissue; MSC, mesenchymal stem cell; OA, osteoarthritis; PRISMA, Preferred Reporting Items for Systematic Reviews and Meta-Analyses; RCT, randomized controlled trial; RoB 2, Cochrane Risk of Bias tool version 2; ROBINS-I, Risk Of Bias In Non-randomized Studies of Interventions; SVF, stromal vascular fraction; SWiM, Synthesis Without Meta-analysis; TEAE, treatment-emergent adverse event; TGF-β1, transforming growth factor beta 1; UCB-MSC, umbilical cord blood-derived mesenchymal stem cell; VAS, visual analog scale; WOMAC, Western Ontario and McMaster Universities Osteoarthritis Index.
