# Supplementary material for: MSC–Hydrogel Composite Systems for Knee Cartilage Repair and Osteoarthritis: A Systematic Review
Source: Gels. 2026 Aug 13;12(8):715. doi: 10.3390/gels12080715 (PMC13512485; doi:10.3390/gels12080715)
Supplement: Supplementary file 1 [file gels-12-00715-s001.zip › gels-4405171-Supplementary Checklist S1.pdf]

PRISMA 2020 Checklist

Manuscript: MSC–Hydrogel Composite Systems for Knee Cartilage Repair and Osteoarthritis: A Systematic Review

Journal: Gels | Manuscript ID: gels-4405171 | PROSPERO: CRD420261393525

Note: Locations are provided by manuscript section/subsection and approximate page range in the current MDPI layout version. Page numbering may shift after editorial typesetting.

Section	Topic	Item	PRISMA 2020 checklist item	Location where item is reported
TITLE	Title	1	Identify the report as a systematic review.	Title page / Article title
ABSTRACT	Abstract	2	See the PRISMA 2020 for Abstracts checklist.	Abstract
INTRODUCTION	Rationale	3	Describe the rationale for the review in the context of existing knowledge.	Introduction, final paragraph and preceding rationale
INTRODUCTION	Objectives	4	Provide an explicit statement of the objective(s) or question(s) the review addresses.	Introduction, final paragraph; Abstract, Objectives
METHODS	Eligibility criteria	5	Specify the inclusion and exclusion criteria for the review and how studies were grouped for the syntheses.	Methods, Section 2.2; Table 1 (PICOS framework)
METHODS	Information sources	6	Specify all databases, registers, websites, organisations, reference lists and other sources searched or consulted to identify studies. Specify the date when each source was last searched or consulted.	Methods, Section 2.3
METHODS	Search strategy	7	Present the full search strategies for all databases, registers and websites, including any filters and limits used.	Methods, Section 2.3; search strategy to be provided in Supplementary Material if requested
METHODS	Selection process	8	Specify the methods used to decide whether a study met the inclusion criteria, including how many reviewers screened each record and report, whether they worked independently, and how disagreements were resolved.	Methods, Section 2.4
METHODS	Data collection process	9	Specify the methods used to collect data from reports, including how many reviewers collected data, whether they worked independently, processes for obtaining or confirming data from investigators, and how disagreements were resolved.	Methods, Section 2.5
METHODS	Data items	10a	List and define all outcomes for which data were sought. Specify whether all results compatible with each outcome domain in each study were sought.	Methods, Sections 2.2 and 2.5; Table 1
METHODS	Data items	10b	List and define all other variables for which data were sought, such as participant and intervention characteristics, funding sources, and assumptions/simplifications made.	Methods, Section 2.5
METHODS	Study risk of bias	11	Specify the methods used to assess risk of bias in the included	Methods, Section 2.6; Results, Section

	assessment		studies, including details of the tool(s), how many reviewers assessed each study, whether they worked independently, and how disagreements were resolved.	3.4; Table 3
METHODS	Effect measures	12	Specify for each outcome the effect measure(s), such as risk ratio or mean difference, used in the synthesis or presentation of results.	Methods, Section 2.7; Results, Section 3.5; narrative synthesis without pooled effect estimates
METHODS	Synthesis methods	13a	Describe the processes used to decide which studies were eligible for each synthesis.	Methods, Sections 2.2 and 2.7; Results, Sections 3.1 and 3.5
METHODS	Synthesis methods	13b	Describe any methods required to prepare the data for presentation or synthesis, such as handling of missing summary statistics or data conversions.	Methods, Section 2.7; narrative synthesis reported by outcome domain
METHODS	Synthesis methods	13c	Describe any methods used to tabulate or visually display results of individual studies and syntheses.	Methods, Section 2.7; Tables 2–4; Figure 1
METHODS	Synthesis methods	13d	Describe any methods used to synthesize results and provide a rationale for the choice. If meta-analysis was performed, describe the model(s), methods to identify heterogeneity, and software used.	Methods, Section 2.7; meta-analysis not performed because threshold for comparable outcomes was not met
METHODS	Synthesis methods	13e	Describe any methods used to explore possible causes of heterogeneity among study results.	Methods, Section 2.7; Results, Sections 3.3–3.5; Discussion
METHODS	Synthesis methods	13f	Describe any sensitivity analyses conducted to assess robustness of synthesized results.	Not applicable; no quantitative meta-analysis was performed
METHODS	Reporting bias assessment	14	Describe any methods used to assess risk of bias due to missing results in a synthesis, arising from reporting biases.	Methods, Section 2.7; GRADE assessment included publication/reporting bias considerations; Table 4
METHODS	Certainty assessment	15	Describe any methods used to assess certainty or confidence in the body of evidence for each outcome.	Methods, Section 2.7; Results, Section 3.6; Table 4
RESULTS	Study selection	16a	Describe the results of the search and selection process, from the number of records identified in the search to the number of studies included in the review, ideally using a flow diagram.	Results, Section 3.1; Figure 1 (PRISMA 2020 flow diagram)
RESULTS	Study selection	16b	Cite studies that might appear to meet inclusion criteria but were excluded, and explain why they were excluded.	Results, Section 3.1; exclusion reasons summarized in Figure 1
RESULTS	Study characteristics	17	Cite each included study and present its characteristics.	Results, Section 3.2; Table 2
RESULTS	Risk of bias in studies	18	Present assessments of risk of bias for each included study.	Results, Section 3.4; Table 3
RESULTS	Results of individual studies	19	For all outcomes, present for each study summary statistics and effect estimates, preferably using structured tables or plots.	Results, Section 3.5; Tables 2 and 4

RESULTS	Results of syntheses	20a	For each synthesis, briefly summarize the characteristics and risk of bias among contributing studies.	Results, Sections 3.2–3.6; Tables 2–4
RESULTS	Results of syntheses	20b	Present results of all statistical syntheses conducted. If meta-analysis was done, present summary estimate and precision; if comparing groups, describe direction of effect.	Results, Section 3.5; no pooled meta-analysis conducted
RESULTS	Results of syntheses	20c	Present results of all investigations of possible causes of heterogeneity among study results.	Results, Sections 3.3 and 3.5; Discussion
RESULTS	Results of syntheses	20d	Present results of all sensitivity analyses conducted to assess robustness of synthesized results.	Not applicable; no quantitative sensitivity analysis was conducted
RESULTS	Reporting biases	21	Present assessments of risk of bias due to missing results for each synthesis assessed.	Results, Section 3.6; Table 4
RESULTS	Certainty of evidence	22	Present assessments of certainty or confidence in the body of evidence for each outcome assessed.	Results, Section 3.6; Table 4
DISCUSSION	Discussion	23a	Provide a general interpretation of the results in the context of other evidence.	Discussion, Section 4
DISCUSSION	Discussion	23b	Discuss any limitations of the evidence included in the review.	Discussion, Section 4; Conclusions
DISCUSSION	Discussion	23c	Discuss any limitations of the review processes used.	Discussion, Section 4; Methods, Sections 2.3–2.7
DISCUSSION	Discussion	23d	Discuss implications of the results for practice, policy, and future research.	Discussion, Section 4; Conclusions
OTHER INFORMATION	Registration and protocol	24a	Provide registration information for the review, including register name and registration number, or state that the review was not registered.	Abstract, Methods Section 2.1: PROSPERO CRD420261393525
OTHER INFORMATION	Registration and protocol	24b	Indicate where the review protocol can be accessed, or state that a protocol was not prepared.	Methods, Section 2.1; PROSPERO registration cited
OTHER INFORMATION	Registration and protocol	24c	Describe and explain any amendments to information provided at registration or in the protocol.	Not reported / no amendments specified
OTHER INFORMATION	Support	25	Describe sources of financial or non-financial support for the review, and the role of the funders or sponsors.	Funding statement
OTHER INFORMATION	Competing interests	26	Declare any competing interests of review authors.	Conflicts of Interest statement
OTHER INFORMATION	Availability of data, code and other materials	27	Report which materials are publicly available and where they can be found, such as template data collection forms, extracted data, analytic code, and other materials.	Data Availability Statement; extracted data are available within the article and tables

Prepared for submission with the revised manuscript gels-4405171.
