# Supplementary material for: MSC–Hydrogel Composite Systems for Knee Cartilage Repair and Osteoarthritis: A Systematic Review
Source: Gels. 2026 Aug 13;12(8):715. doi: 10.3390/gels12080715 (PMC13512485; doi:10.3390/gels12080715)
Supplement: Supplementary file 1 [file gels-12-00715-s001.zip › gels-4405171-Supplementary Tables.pdf]

Supplementary Table S1. Complete database-specific search strategies used for study identification.

Database	Search strategy
PubMed/MEDLINE	("mesenchymal stem cell*" OR MSC OR "mesenchymal stromal cell*") AND (hydrogel* OR scaffold* OR biomaterial*) AND (knee OR cartilage OR chondral OR osteochondral OR osteoarthritis)
Embase	('mesenchymal stem cell'/exp OR 'mesenchymal stromal cell':ti,ab OR MSC:ti,ab) AND ('hydrogel'/exp OR hydrogel*:ti,ab) AND ('knee'/exp OR cartilage:ti,ab OR chondral:ti,ab OR osteochondral:ti,ab OR osteoarthritis:ti,ab)
Scopus	TITLE-ABS-KEY ("mesenchymal stem cell*" OR MSC OR "mesenchymal stromal cell*") AND TITLE-ABS-KEY (hydrogel* OR scaffold* OR biomaterial*) AND TITLE-ABS-KEY (knee OR cartilage OR chondral OR osteochondral OR osteoarthritis)
Web of Science	TS=("mesenchymal stem cell*" OR MSC OR "mesenchymal stromal cell*") AND TS=(hydrogel* OR scaffold* OR biomaterial*) AND TS=(knee OR cartilage OR chondral OR osteochondral OR osteoarthritis)
Cochrane CENTRAL	("mesenchymal stem cells" OR MSC) AND (hydrogel OR scaffold) AND (knee OR cartilage OR osteoarthritis)

Search date: April–May 2026.
Restrictions: No restrictions on publication year, language, or study design.

Supplementary Table S2. Technical Characteristics of Included MSC–Hydrogel Studies

Study	MSC Source	Hydrogel	Cell Dose	Delivery Method	Defect Size	Follow-up
Park 2017	UCB-MSC	4% HA	7.5×10^6	Implantation	NR	7 years
Song 2020	UCB-MSC	4% HA	7.5×10^6	Implantation	NR	≥2 years
Suh 2021	UCB-MSC	4% HA	7.5×10^6	Implantation	NR	18 months
Lim 2021	UCB-MSC	4% HA	7.5×10^6	Implantation	Large full-thickness defects	5 years
Toktarov 2022	Sy-MSC	Heparin-fibrin + TGF-β1/BMP-4	1.15–2.0 $\times 10^7$	Arthroscopic implantation	NR	6 months
Kim 2023	UCB-MSC	4% HA	7.5×10^6	Implantation	NR	12.6 months
Lee 2025	UCB-MSC	4% HA	7.5×10^6	Implantation	NR	≥2 years
Jung 2025	UCB-MSC	4% HA	7.5×10^6	Implantation	Mean ~6.3 cm^2 threshold reported	1 year
